# Hydroxycarboxylic acid receptors are essential for breast cancer cells to control their lipid/fatty acid metabolism

**DOI:** 10.18632/oncotarget.3565

**Published:** 2015-03-14

**Authors:** Claudia Stäubert, Oliver Jay Broom, Anders Nordström

**Affiliations:** ^1^ Swedish Metabolomics Centre, Department of Forest Genetics and Plant Physiology, Swedish University of Agricultural Sciences, Umeå, Sweden; ^2^ Department of Molecular Biology, Umeå University, Umeå, Sweden; ^3^ Institute of Biochemistry, Faculty of Medicine, University of Leipzig, Leipzig, Germany

**Keywords:** hydroxycarboxylic acid receptors, cancer metabolism, metabolite-sensing GPCRs, GPR81, GPR109a

## Abstract

Cancer cells exhibit characteristic changes in their metabolism with efforts being made to address them therapeutically. However, targeting metabolic enzymes as such is a major challenge due to their essentiality for normal proliferating cells. The most successful pharmaceutical targets are G protein-coupled receptors (GPCRs), with more than 40% of all currently available drugs acting through them.

We show that, a family of metabolite-sensing GPCRs, the Hydroxycarboxylic acid receptor family (HCAs), is crucial for breast cancer cells to control their metabolism and proliferation.

We found HCA_1_ and HCA_3_ mRNA expression were significantly increased in breast cancer patient samples and detectable in primary human breast cancer patient cells. Furthermore, siRNA mediated knock-down of HCA_3_ induced considerable breast cancer cell death as did knock-down of HCA_1_, although to a lesser extent. Liquid Chromatography Mass Spectrometry based analyses of breast cancer cell medium revealed a role for HCA_3_ in controlling intracellular lipid/fatty acid metabolism. The presence of etomoxir or perhexiline, both inhibitors of fatty acid β-oxidation rescues breast cancer cells with knocked-down HCA_3_ from cell death.

Our data encourages the development of drugs acting on cancer-specific metabolite-sensing GPCRs as novel anti-proliferative agents for cancer therapy.

## INTRODUCTION

Ever since Warburg's discovery of aerobic glycolysis as a metabolic hallmark of cancer cells, extensive studies have increased our understanding of cancer cell metabolism [1, 2]. Characteristic metabolic changes, besides aerobic glycolysis have been identified including, increased lactate production, glutamine metabolism, and fatty acid synthesis, coupled with decreased fatty acid oxidation [1, 2]. Cancer-specific up-regulated enzymes involved in central metabolic pathways have been identified, and have come into focus as targets for cancer therapy [3-5]. However, because all cells depend on the same central metabolic pathways, one main obstacle is the toxicity of drugs acting upon those enzymes [3-5].

**Figure 1:**
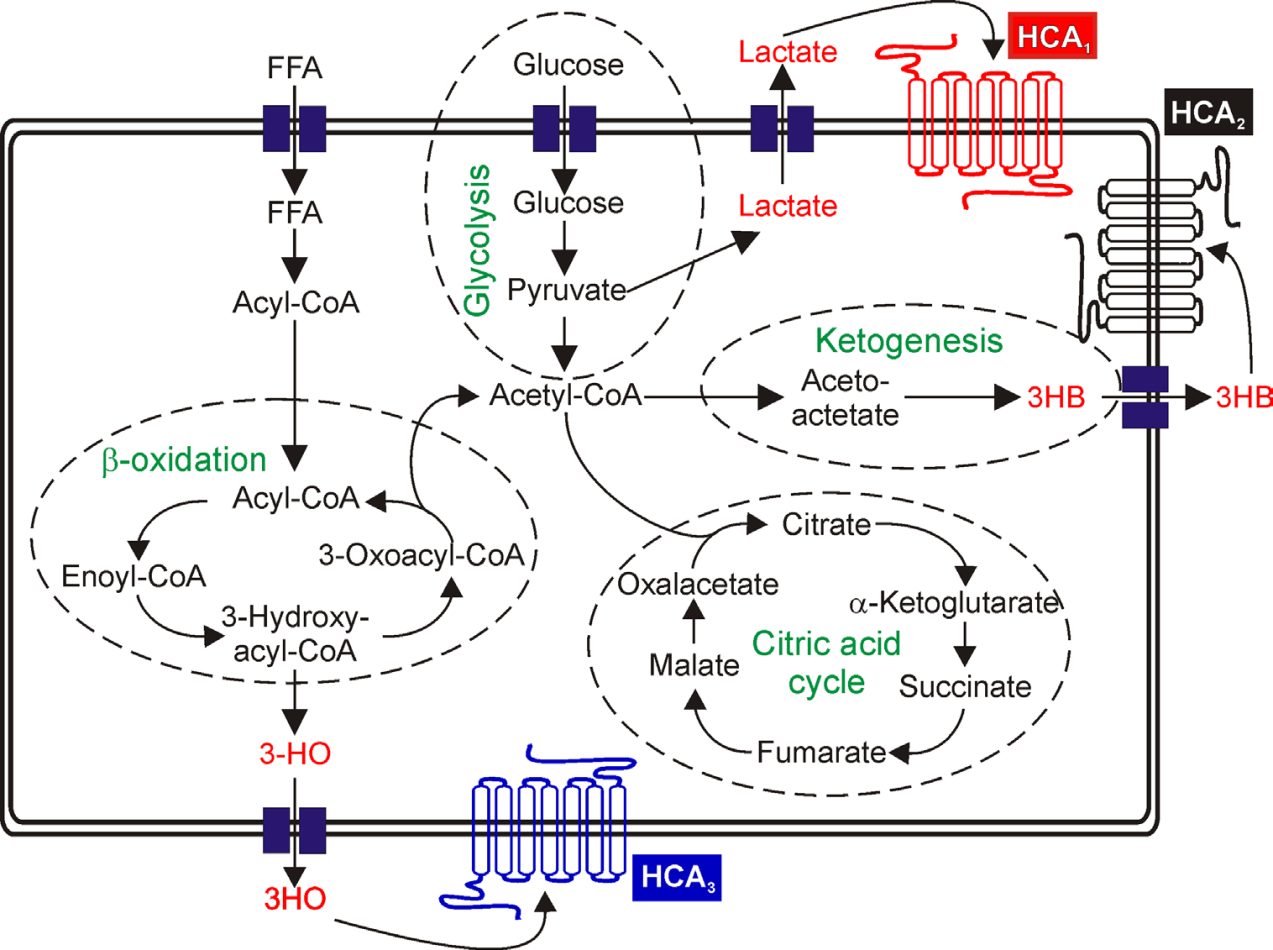
Schematic overview of HCA agonist generating metabolic pathways Lactate, the endogenous agonist of HCA_1_, is an indicator for increased rates of glycolysis. Excess acetyl-CoA is converted to ketone bodies, one of which is 3HB - the endogenous agonist of HCA_2_ and 3HO, agonist of HCA_3_ is an intermediate of FAO. FFA: free fatty acid.

G protein-coupled receptors (GPCRs) constitute the largest family of transmembrane receptors, transduce diverse extracellular signals inside the cell and represent one of the major pharmaceutical targets [6, 7]. Recently, a growing number of so far orphan GPCRs, have been shown to be activated by metabolic intermediates or energy substrates [8]. The HCA family of receptors consists of three members that are mainly expressed in adipocytes [9, 10]. Activation by their respective agonists inhibits adipocyte lipolysis [9, 10]. HCA_1_ is activated by lactate, a product of glycolysis, the endogenous agonist for HCA_2_ is 3-hydroxybutyrate (3HB), a ketone body and for HCA_3_, 3-hydroxyoctanoate (3HO), an intermediate of fatty acid β-oxidation (FAO) (Figure 1) [9, 10].

Since HCAs are activated by intermediates of central metabolic processes that are often differentially regulated in cancer cells (e.g. glycolysis), we set out to investigate their potential role for cancer cell proliferation.

Here, we demonstrate that HCA_1_ and HCA_3_ mRNA expression is increased in human breast cancer patient tissue as compared to normal tissue samples, and in primary breast cancer cells. We provide evidence, that HCA_3_ and to a lesser extent HCA_1_, are essential for breast cancer cells to control their lipid/fatty acid metabolism. Cancer cell metabolism is perturbed when cellular transmembrane “metabolic surveillance”, through namely HCA_1_ and HCA_3_, is abrogated causing a decrease in viability and/or cell death. Thus, HCA_1_ and HCA_3_ constitute potential targets for therapeutic intervention in cancer.

## RESULTS

### Breast cancer patient tissue exhibits higher HCA mRNA expression levels when compared to normal breast tissue

**Figure 2:**
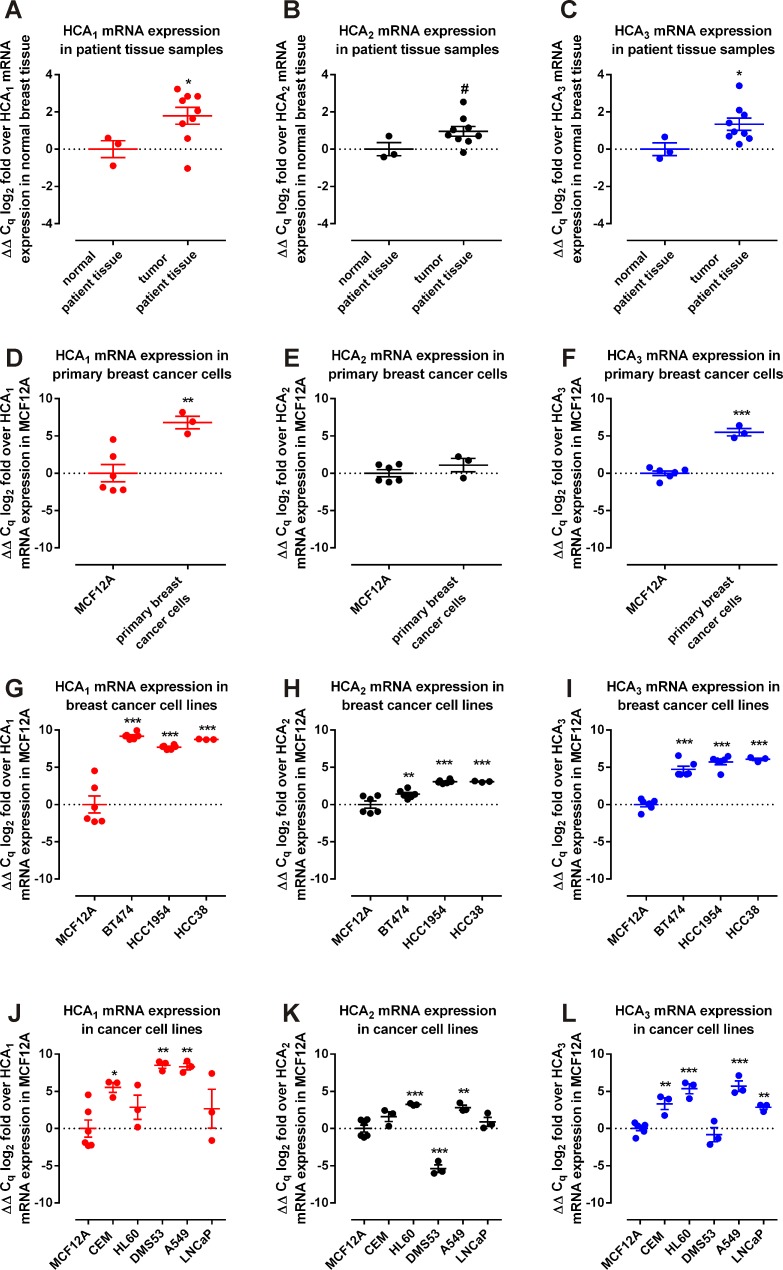
HCAs are overexpressed in human patient breast cancer tissue, primary breast cancer cells and breast cancer cell lines (A-C) Expression of HCAs in breast cancer (n = 9) versus normal (n = 3) patient tissue (two-tailed unpaired t-test, Welch's correction). (D-F) Expression of HCAs in primary human breast cancer cells (n = 3) versus non-tumorigenic epithelia breast cells MCF12A (two-tailed unpaired t-test, Welch's correction). (G-I) HCA expression in breast cancer cells BT-474, HCC1954 and HCC38 versus non-tumorigenic epithelia breast cells MCF12A (n = 6 for all except for HCC38 n = 3, ordinary One-Way ANOVA, Dunnett's multiple comparisons test). (J-L) mRNA expression of HCA in two leukemia cell lines (CEM, HL60), two lung cancer cell lines (DMS53, A549) and one prostate cancer cell line (LNCaP) versus non-tumorigenic epithelia breast cells MCF12A (n = 3 except for MCF12A n = 6, ordinary One-Way ANOVA, Dunnett's multiple comparisons test). Data is shown as mean ± SEM. ^#^ P ≤ 0.1* P ≤ 0.05; ** P ≤ 0.01; *** P ≤ 0.001.

Since a relevance of HCAs for cancer cell metabolism can only be assumed if they are expressed in human cancer patient tissue, we first analyzed the mRNA expression levels of HCA_1_, HCA_2_ and HCA_3_ in eight different cancers versus the respective normal tissues. For this purpose we used the Cancer and Normal TissueScan^TM^ Cancer Survey cDNA qPCR Array – I (CSRT501) (Origene) which contains tissue cDNAs that are synthesized from high quality total RNAs of pathologist-verified tissues, normalized and validated with β-actin in two sequential qPCR analyses, and are provided with clinical information and QC data. HCA_2_ and HCA_3_ mRNA expression was significantly higher in colon cancer and HCA_2_ was lower in kidney, slightly lower in lung and slightly increased in ovarian cancer samples (Figure S1). However, the strongest differential mRNA expression of HCA_1_ (Figure 2A), HCA_2_ (Figure 2B) and HCA_3_ (Figure 2C) was detected in breast cancer patient versus normal tissue samples, with HCA_1_ showing about 5-fold, HCA_2_ about 2-fold and HCA_3_ about 3-fold higher mRNA expression levels (Figure 2A-C).

### HCA_1_ and HCA_3_ transcripts are expressed in primary breast cancer cells

Following these results, we obtained primary breast cancer cells from three different patients and analyzed HCA mRNA expression. We found HCA_1_ mRNA expression more than 100-fold (Figure 2D), HCA_2_ mRNA expression unchanged (Figure 2E) and HCA_3_ mRNA expression about 50-fold higher (Figure 2F) in the primary breast cancer cells when compared to HCA mRNA expression in the non-tumorigenic epithelial breast cell line MCF12A.

### Higher HCA mRNA expression in breast cancer cell lines

Next, we set out to analyze the HCA mRNA expression in the breast cancer cell lines BT-474 (progesterone receptor (PR) positive, estrogen receptor (ER) positive, HER2/neu positive), HCC1954 (PR negative, ER negative, HER2/neu positive) and HCC38 (PR negative, ER negative, HER2/neu negative) versus the non-tumorigenic epithelial breast cell line MCF12A. Compared to MCF12A, the mRNA levels of all three HCAs were significantly higher in all three breast cancer cell lines (Figure 2G-I).

### HCA mRNA expression in other cancer cell lines

We further assessed HCA mRNA expression in two leukemia, two lung cancer and one prostate cancer cell lines relative to HCA expression in MCF12A (Figure 2J-L). HCA_1_ mRNA expression was significantly higher in the lung cancer cell lines A549 and DMS53 as well as in the acute lymphoblastic leukemia cell line CEM (Figure 2J). Expression of HCA_2_ transcripts was found significantly higher in the acute promyelocytic leukemia cell line HL60 and the lung cancer cell line A549, whereas expression in DMS53 cells was lower when compared to HCA_2_ mRNA expression in MCF12A cells (Figure 2K). All cancer cell lines except DMS53 showed an increased HCA_3_ mRNA expression (Figure 2L). Presence of HCA_2_ and HCA_3_ mRNA transcripts has been demonstrated before in LoVo colorectal adenocarcinoma cells [11].

### Comparison of HCA mRNA expression levels in normal tissue relative to expression in breast

In Table S1 we provide the information given by Origene for all normal tissue samples included in the Cancer and Normal TissueScan^TM^ Cancer Survey cDNA qPCR Array – I (CSRT501) (Origene). Analyzing these samples using RT-qPCR we found highest HCA_1_ mRNA in breast tissue (Figure S2A). No HCA_1_ expression was detectable in normal colon, kidney and liver tissue samples. HCA_2_ mRNA expression could be shown for all normal tissue samples with highest level of expression in kidney and liver (Figure S2B). Transcripts of HCA_3_ were also detectable in all normal tissue samples with highest expression in kidney, liver and lung (Figure S2C).

### HCA mRNA expression levels relative to HCA_1_ in cancer cell lines

Moreover, we analyzed our RT-qPCR data obtained from all 9 cell lines regarding the expression of HCA_2_ and HCA_3_ relative to HCA_1_ to provide information about the relative abundance of HCA transcripts in comparison to each other. In MCF12A, (Figure S2D), HCC1954 (Figure S2F), HL60 (Figure S2I) and LNCaP cells HCA_2_ and HCA_3_ transcripts are more abundant than HCA_1_ transcripts. The opposite is true for BT-474 (Figure S2E) and DMS53 (Figure S2J) cells and no difference in HCA transcript abundance is found in HCC38 (Figure S2G), CEM (Figure S2H) and A549 (Figure S2K) cells.

### HCA agonist stimulation induces a decrease in intracellular cAMP levels in BT-474

**Figure 3:**
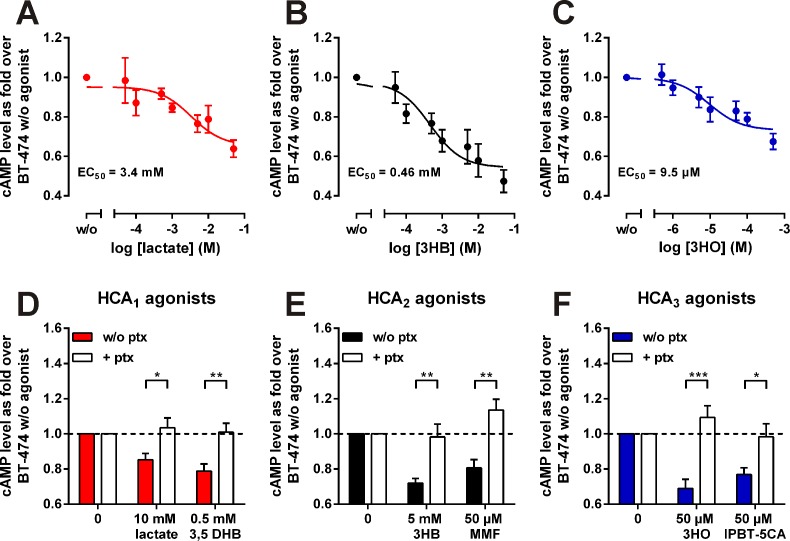
Lactate, 3-hydroxybutyrate (3HB) and 3-hydroxyoctanoate (3HO) activate endogenous HCA receptors in BT-474 cells in a pertussis toxin sensitive manner (A-C) HCA agonist-mediated inhibition of forskolin-induced cAMP production in BT-474 cells was determined using the AlphaScreen® cAMP Assay Kit. The cyclic AMP level of cells stimulated with 10 μM forskolin in absence of agonist was set 1. EC_50_ values were determined from concentration-response curves of agonists using GraphPad Prism (n = 3). (D-F) Agonist-induced decrease in intracellular cAMP levels is pertussis toxin sensitive (n = 4). 3,5 DHB: 3,5-dihydroxybenzoic acid, MMF: mono-methyl fumarate, IPBT-5CA (IBC-293): 1-(1-Methylethyl)-1H-benzotriazole-5-carboxylic acid. All data is shown as mean ± SEM. p-values were determined using a two-tailed unpaired t-test. * P ≤ 0.05; ** P ≤ 0.01; *** P ≤ 0.001.

All members of the HCA receptor family are Gα_i_-coupled receptors [10]. Thus, agonist-mediated receptor activation induces inhibition of adenylyl cyclase and the intracellular cAMP level is subsequently reduced. We performed cAMP assays in the presence of 10 μM forskolin and increasing concentrations of lactate, 3HB or 3HO in BT-474 (Figure 3A-B). Thereby we were able to determine an EC_50_ of 3.4 mM for lactate, 0.46 mM for 3HB and 9.5 μM for 3HO (Figure 1D-F). Lactate has been reported to activate human HCA_1_ with an EC_50_ in the range of 1.3 - 5 mM [12-14]. The EC_50_ value of 3HB activating HCA_2_ has been reported as 0.7 mM [15] and of 3HO activating HCA_3_ as 8 μM [16]. Accordingly, our data strongly suggests the presence of functional HCA_1_, HCA_2_ and HCA_3_ receptor protein in BT-474 cells. To exclude that the inhibitory effects on cAMP levels exerted by lactate, 3HB and 3HO are unspecific effects of high metabolite concentrations, we tested their sensitivity to pertussis toxin. Pertussis toxin is an inhibitor of the Gα_i_ protein and blocked both endogenous (lactate, 3HB and 3HO) and synthetic HCA agonist-induced decrease in intracellular cAMP levels in BT-474 cells (Figure 3D-F). 3,5 dihydroxybenzoic acid is a specific HCA_1_ agonist [17], HCA_2_ is known to be activated by the antipsoriatic drug monomethyl fumarate [18] and IPBT-5CA (IBC-293, 1-(1-methylethyl)-1H-benzotriazole-5-carboxylic acid) is a selective HCA_3_ agonist [19, 20].

### siRNA directed against HCA_1_ and HCA_3_ decreases the viability of breast cancer cell lines but not of MCF12A and HEK293T

**Figure 4:**
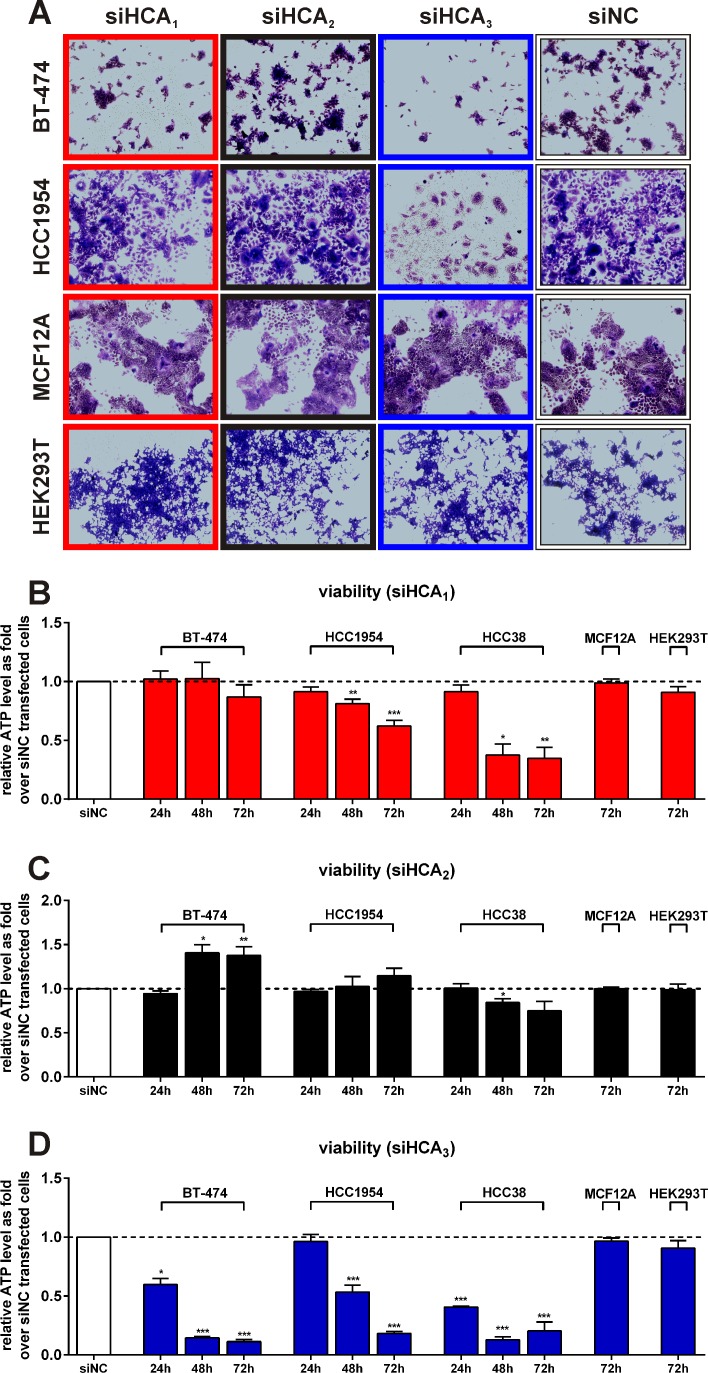
Effect of HCA_1_, HCA_2_ and HCA_3_ siRNA mediated knock-down on BT-474, HCC1954, HCC38, MCF12A and HEK293T cell viability (A) Crystal violet staining of BT-474, HCC1954 MCF12A and HEK293T cells transfected with siRNA directed against HCA_1_, HCA_2_ and HCA_3_ versus scrambled negative control (siNC) after 48h. Cell viability of siHCA_1_ (B), siHCA_2_ (C) and siHCA_3_ (D) versus siNC transfected BT-474, HCC1954 (both: 24h: n = 3, 48h: n = 4, 72h: n = 6), HCC38 (24h, 48h, 72h: each n = 3) MCF12A and HEK293T (both 72h: n = 3) cells. All data is shown as mean ± SEM. p-values were determined using a two-tailed unpaired t-test. * P ≤ 0.05; ** P ≤ 0.01; *** P ≤ 0.001.

To assess whether the HCAs are essential for breast cancer cell line proliferation we performed siRNA-mediated knock-down experiments. Microscopy images of crystal violet-stained cells revealed that knock-down of HCA_1_ slightly and of HCA_3_ considerably, decreased viability in both BT-474 and HCC1954 whereas no effect was observed for MCF12A and HEK293T cells (Figure 4A). These qualitative results were further confirmed by quantification of cell viability through measurement of the relative ATP level. The knock-down efficiency as well as the co-regulation of related HCA mRNA expression, was monitored using reverse-transcriptase quantitative PCR (RT-qPCR).

For all experiments, we used pooled siRNA containing one third of each A, B and C siRNA provided from OriGene (Table S2). However, we also measured the viability of BT-474 and HCC1954 cells upon the transfection of each individual siRNA. Thereby, we were able to detect a significant decrease in viability of both cell lines 72h after transfection with siHCA_1_-A and siHCA_1_-C, or with siHCA_3_-B and siHCA_3_-C (Figure S3).

An 80% knock-down of HCA_1_(Figure S4A) caused a 20% and 40% decrease in HCC1954 viability after 48h and 72h, respectively, whereas it had no significant influence on BT-474 viability (Figure 4B). HCA_1_ knock-down was accompanied by an increased mRNA expression of HCA_2_ and HCA_3_ (Figure S4A). 48h after siHCA_1_ transfection HCC38 viability was already about 60% reduced. But, 72h after transfection with siHCA_1_ no effect on viability was observed for cells that do not express HCA_1_, specifically MCF12A and HEK293T (Figure 4B).

In contrast, the viability of BT-474 cells exhibiting a 60-70% knock-down of HCA_2_ (Figure S4B) was significantly increased, had no effect on HCC1954, MCF12A and HEK293T viability and induced a significant reduction in viability in HCC38 cells (Figure 4C).

The cell viability of the BT-474 cells was 40%, 80% and 90% reduced 24h, 48h and 72h after siHCA_3_ transfection, and for the HCC1954 cells, 50% and 80% after 48h and 72h, respectively (Figure 4D). The viability of the siHCA_3_ transfected HCC38 cells was already after 24h 60% lower when compared to siNC transfected HCC38 cells and only 10-20% of cells were still alive after 48h and 72h (Figure 4D).

**Figure 5:**
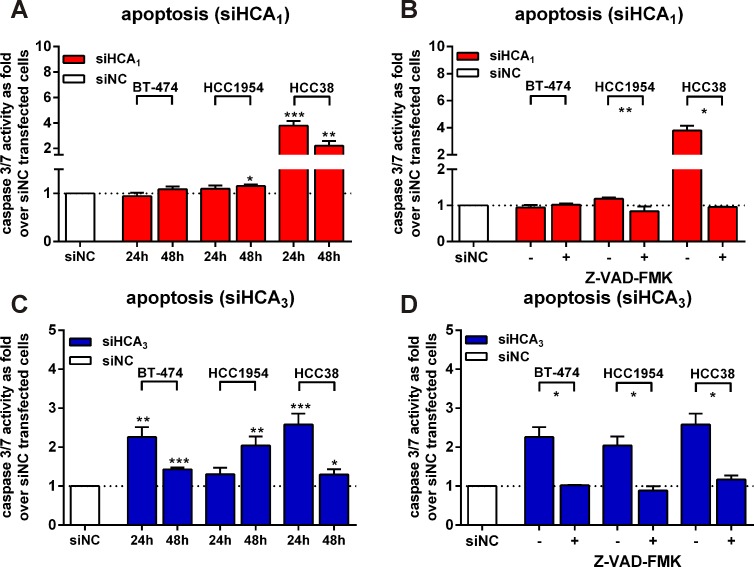
Knock-down of HCA_1_ and HCA_3_ induces apoptosis in breast cancer cell lines through caspase 3/7 activation that is diminished with the pan-caspase inhibitor Z-VAD-FMK (A) Caspase 3/7 activity in siHCA_1_ versus siNC transfected BT-474, HCC1954 and HCC38 cells. (B) Z-VAD-FMK blocks siHCA_1_ induced apoptosis in HCC1954 and HCC38 cells. (C) siHCA_3_ transfection induces caspase 3/7 activity in all three breast cancer cell lines. (D) siHCA_3_ induced caspase 3/7 activity is diminished in the presence of 30 μM Z-VAD-FMK (24h after transfection: BT-474, HCC38; 48h post-transfection: HCC1954). All data is shown as mean ± SEM of three independent experiments carried out in triplicates. p-values were determined using a two-tailed unpaired t-test. * P ≤ 0.05; ** P ≤ 0.01; *** P ≤ 0.001.

The HCA_3_ knock-down could not reliably be determined when experiments were carried out in presence of 2.5% fetal bovine serum (FBS) which was used to ensure high transfection efficiency (Figure S4C). The knock-down of HCA_3_ in breast cancer cells causes induction of apoptosis in those cells (Figure 5C) which in turn leads to changes in reference gene expression, thus preventing the reliable determination of ΔΔC_q_. However, with serial reduction of HCA_3_ specific siRNA and performing the experiments in the presence of 10% FBS, transfection efficiency was decreased and viability less effected (Figure S5A-D) thereby allowing detection of 30-40% and 20-70% siHCA_3_-mediated decrease in HCA_3_ mRNA levels in BT-474 and HCC1954, respectively (Figure S5E-F). Further, we assessed HCA cell surface expression in transiently HCA overexpressing HEK293T cells co-transfected with either respective HCA-specific siRNA or siNC using an indirect cellular ELISA (Figure S6A-C). 24h after co-transfection with HA-tagged HCA-encoding plasmid and siRNA we could detect an approximate 50% decrease in HA-tagged HCA_1_ cell surface expression (Figure S6A), 40% less HA-tagged HCA_2_ receptor on the cell surface (Figure S6B) and approximately 60% reduction of the HA-tagged HCA_3_ cell surface expression in HEK293T cells (Figure S6C).

### Transiently HCA_1_ and HCA_3_ overexpressing BT-474 and HCC1954 show increased viability when compared to mock-transfected cells

We next tested the influence of transient overexpression of HCA_1_, HCA_2_ and HCA_3_ on the BT-474 and HCC1954 cell viability. Transiently transfected, HCA_1_-overexpressing BT-474 and to lesser extent HCC1954 exhibited an increased cell viability when compared to cells transfected with empty vector (Figure S6D). The cell viability of HCA_2_ overexpressing HCC1954 was reduced after 72h whereas no effect was observed in BT-474 cells (Figure S6E). An increased viability in transiently HCA_3_ overexpressing BT-474 and HCC1954 cells was observed (Figure S6F).

### Knock-down of HCA_1_ and HCA_3_ induces apoptosis in breast cancer cell lines through caspase 3/7 activation that is diminished in the presence of the pan-caspase inhibitor Z-VAD-FMK

Subsequently, we analyzed whether the observed siHCA_1_- and siHCA_3_-induced loss in breast cancer viability was due to apoptosis. Therefore, we measured caspase 3/7 activity 24h and 48h after siHCA_1_ or siHCA_3_ transfection in all three breast cancer cell lines. Increased caspase 3/7 activity was detectable in siHCA_1_ transfected HCC1954 cells after 48h and in HCC38 cells after 24h and 48h (Figure 5A). In the presence of 30 μM of the cell-permeable pan-caspase inhibitor Z-VAD-FMK the signal was completely diminished (Figure 5B). The HCA_3_ knock-down induced apoptosis in all three breast cancer cell lines, where in the BT-474 and HCC38 cells the obtained caspase 3/7 activity was highest 24h, but in HCC1954 48h after siHCA_3_ transfection (Figure 5C). Caspase 3/7 activity could not be detected in siHCA_3_ transfected breast cancer cells when Z-VAD-FMK was present (Figure 5D).

### HCA_3_ knock-down causes a dysregulation of the lipid/fatty acid metabolism

We hypothesize that HCA_3_ is important for controlling the balance of lipid/fatty acid metabolism in breast cancer cells. To test our hypothesis, we used Liquid-Chromatography Mass Spectrometry (LC-MS) to analyze compounds related to lipid/fatty acid metabolism in the medium of siHCA_3_ versus siNC transfected BT-474 cells (Table S3).

**Figure 6:**
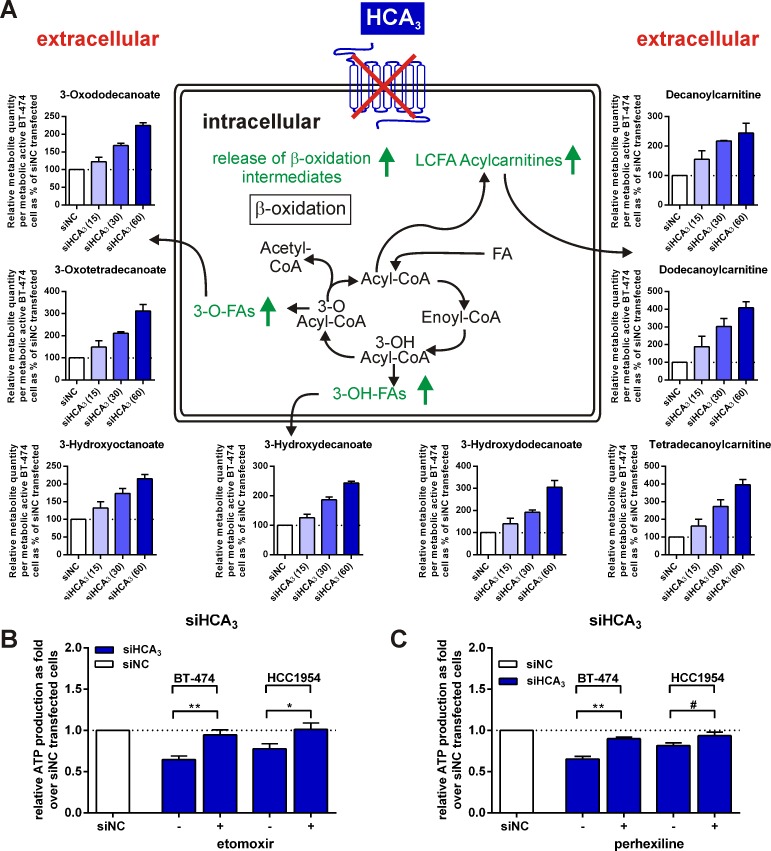
Extracellular concentration of FAO intermediates is increased for BT-474 with knocked-down HCA_3_ compared to siNC transfected cells and viability is rescued in presence of FAO inhibitors (A) Metabolites differing in medium of siHCA_3_ versus siNC transfected BT-474 cells point towards an increased FAO rate in cells with knocked-down HCA_3_. Total siRNA concentration in each well was 60 nM. Concentration of HCA_3_-specific siRNA is specified on the x-axis inside the parentheses. FAO intermediates were determined using LC-MS (n = 2) and normalized to viable cells (shown in Figure S5C). The data shown reflects relative metabolite quantity produced per metabolically active cells. The value determined for medium of siNC transfected BT-474 was set 100%. Peak height of compounds relative to siNC transfected cells are stated in Table S3. BT-474 and HCC1954 cell viability with knocked-down HCA_3_ is rescued upon co-administration of (B) 30 μM etomoxir (n = 4) or (C) 2.5 μM perhexiline (n = 3), both inhibitors of FAO. All data is shown as mean ± SEM. ^#^ P ≤ 0.1* P ≤ 0.05; ** P ≤ 0.01.

The compound quantity measured as the chromatographic peak height, which is proportional to the metabolite concentration (Table S3), and the cell viability, were determined (Figure S6C). BT-474 cells with only a 30% reduced HCA_3_ mRNA level (Figure S6E) and approximately a 25% decreased viability (Figure S6C), take up significantly more lysophosphatidylcholines, reflecting a higher metabolic demand for lipids (Table S3). Moreover, higher levels of C_10_-, C_12_- and C_14_-carnitine were detected in the medium of siHCA_3_ transfected BT-474 cells whereas the short-chain carnitines were unaffected (Figure 6A, Table S3). Furthermore, FAO intermediates such as 3HO, 3-hydroxydecanoate and 3-hydroxydodecanoate as well as 3-oxodocanoate and 3-oxotetradecanoate are liberated to a higher extent from BT-474 with knocked-down HCA_3_ (Table S3). These metabolites were not detectable in the medium alone, thus leading us to the assumption that they are exclusively produced by metabolically active, viable cells. We normalized the obtained metabolite data for these compounds (Table S3) to the cell viability data determined in parallel (Figure S6C), resulting in a metabolite concentration produced per metabolically active cell (Figure 6A). Similarly, because of their lower detection in the medium alone, we proceeded with the C_10_-, C_12_- and C_14_-carnitines after subtraction of the relative quantity of these carnitines detected in medium alone from the raw data counts (Figure 6A). This data revealed a highly increased, siHCA_3_ concentration-dependent production/release of FAO intermediates and carnitines per viable cell (Figure 6A).

### Administration of etomoxir or perhexiline, both inhibitors of FAO, protect BT-474 and HCC1954 cells from siHCA_3_ induced loss in viability in cells

To further analyze the role of FAO in relation to HCA_3_, we tested whether inhibitors of FAO could rescue the siHCA_3_-induced loss of viability in BT-474 and HCC1954. In the presence of etomoxir or perhexiline, both O-carnitine palmitoyltransferase (CPT) inhibitors, no siHCA_3_-induced loss of viability could be observed in BT-474 and HCC1954 cells 24h and 48h after transfection, respectively (Figure 6B, 6C).

## DISCUSSION

Cancer cell metabolism has gained increasingly attention as being a target for therapeutic intervention, with numerous studies contributing to our understanding of metabolic changes in tumor cells [1-5]. Enzymes involved in central metabolic pathways that are specifically up-regulated in cancer, have come into focus as targets for the development of chemotherapeutic drugs [3-5]. The main difficulty encountered is the fact that most of the central metabolic enzymes are also essential for normal proliferating cells. Consequently, drugs acting upon metabolic enzymes are likely to exhibit unwanted side-effects in normal tissue. In the present study, we show that cancer metabolism can be targeted through the metabolite-sensing HCAs. HCAs belong to the GPCR family, which constitutes a major pharmaceutical target with more than 40% of all currently available drugs acting through them [6, 7]. One advantage of targeting cancer metabolism through the transmembrane-spanning GPCRs is that drug-delivery through the membrane to the interior of a cell becomes unnecessary.

In our study, we demonstrate that HCAs are expressed in breast cancer patient tissue, primary breast cancer patient cells and three different breast cancer cell lines (Figure 2). Very recently, HCA_1_ expression has been demonstrated in several cancer cell types, including colon, lung and breast [21] which is in line with the HCA_1_ expression we observe (Figure 2, S1). Here, we show that HCA_1_ is necessary for survival of the HER2-positive breast cancer cell line HCC1954 and the triple-negative HCC38 cells (Figure 4B). Loss of HCA_1_ in those breast cancer cells leads to a loss in viability through apoptosis (Figure 5A). In mice, HCA_1_ mediates anti-lipolytic effects in an insulin dependent manner [13] and regulation of the lipogenic/lipolytic balance is essential for HER2 overexpressing cancer cells to ensure rapid proliferation [5]. Due to a high rate of glycolysis, cancer cells produce high levels of lactate which is exported out of the cell (Figure 1). There, sufficiently high lactate concentrations activate HCA_1_ which subsequently causes inhibition of lipolysis and FAO and as recently shown an increase in mRNA expression levels of genes critical for lactate transport and metabolism [21]. We show, that knock-down of HCA_1_ is accompanied by an increased mRNA expression of HCA_2_ and HCA_3_ (Figure S4A) implying a role for the related HCA family members as secondary metabolic surveillance to monitor cellular metabolic shifts. We suggest that inhibition of FAO/lipolysis through lactate activated HCA_1_ most likely facilitates a higher rate of fatty acid synthesis and lipogenesis, thereby enabling a higher rate of proliferation.

In contrast, the question arises of why HCA_2_ knock-down causes an increase in proliferation of breast cancer cells and HCA_2_ activation suppresses tumor growth as recently shown for colon and mammary tumors [11, 22-24]? Assuming a scenario in which cancer cells shunt glucose to anabolic processes rather than oxidizing it for ATP production, the energy demand has to be satisfied through other pathways such as FAO [2]. By increased lipolysis and subsequent FAO, acetyl-CoA is generated which can enter the citric acid cycle [2]. If the level of acetyl CoA exceeds the capacity of the citric acid cycle, ketone bodies such as 3HB are produced and exported out of the cell where HCA_2_ is activated and subsequently lipolysis is inhibited (Figure 1). By abrogation of this negative feedback mechanism and in the presence of glucose, cancer cells can maintain a high rate of proliferation that is accompanied by ketone body production, which in turn can again be re-utilized as an energy source [25, 26].

Knock-down of HCA_3_ induced cell death in all three breast cancer cell lines BT-474, HCC1954 and HCC38, accompanied by a remarkable decrease in cell viability (Figure 4D, 5C). HCA_3_ is a Gα_i_-coupled receptor that is activated by 3HO [16]. Micromolar levels of 3HO can be detected in patients with FAO disorders, including long-chain, medium-chain and short-chain acyl-CoA dehydrogenase defects [27, 28] as well as in patients with acute diabetic ketoacidosis and in patients that receive a ketogenic diet [16]. Previous studies indicate that elevated levels of medium chain 3-hydroxy fatty acids, including 3HO are indicators of and correlate with increased rates of FAO under physiological and pathophysiological conditions [16, 27-30]. Ahmed *et al*. suggest that HCA_3_ is a sensor for elevated 3HO levels which itself is an indicator of high β-oxidation rates. Activation of HCA_3_ by 3HO would inhibit free fatty acid release and thereby lower their availability for FAO and thus constitutes another negative feedback mechanism to control lipolytic activity [10, 16].

Our data suggests that this negative feedback mechanism mediated by HCA_3_ is crucial for BT-474, HCC1954 and HCC38 cell proliferation and survival. We hypothesize that HCA_3_ has a central role in controlling the balance of lipid/fatty acid metabolism in breast cancer cells. This hypothesis is further supported by our finding of increased levels of C_10_- C_12_- and C_14_-carnitine levels as well as FAO intermediates 3HO, 3-hydroxydecanoate, 3-hydroxydodecanoate, 3-oxodocanoate and 3-oxotetradecanoate in the medium of siHCA_3_ transfected BT-474 (Figure 6A, Table S3). Recent evidence shows that C_10_-carnitine originates from peroxisomal FAO, potentially a compensatory mechanism to metabolize medium- and long-chain fatty acids in cases of mitochondrial FAO overload [31]. Moreover, the extracellular increase of 3-hydroxy- and 3-oxo-FAO intermediates reflects a higher intracellular rate of FAO and especially the extracellular increase of 3HO upon HCA_3_ knock-down strongly supports a role for HCA_3_ in a negative feedback mechanism controlling the FAO rate in BT-474 cells. Further, we show that BT-474 and HCC1954 cell survival with knocked-down HCA3 is rescued in the presence of FAO inhibitors (Figure 6B, 6C) providing further evidence for a critical role of HCA_3_ in controlling the FAO rates in breast cancer cells. In summary, the obtained metabolite data points towards an uncontrolled up-regulated FAO in cells with knocked-down HCA_3_ as the cause for the induced cell death (Figure 4D, 5C, 6A).

We provide evidence that HCAs are important for breast cancer cells to balance their lipid/fatty acid metabolism. Further, we show that cancer cell metabolism can be targeted through GPCRs, thus bypassing direct targeting of intracellular metabolic enzymes. Agonists and antagonists acting at cancer-specific overexpressed metabolite-sensing GPCRs therefore have potential as anti-cancer drugs by perturbing the control of cancer cell metabolism.

## MATERIALS AND METHODS

### Cell lines

Human breast cancer cell lines BT-474 (ATCC® HTB-20™), HCC1954 (ATCC® CRL-2338™) and HCC38 (ATCC® CRL-2314™), the non-tumorigenic epithelial breast cell line MCF12A (ATCC® CRL-10782™), the lung cancer cell lines A549 (ATCC® CCL-185™) and DMS53 (ATCC® CRL-2062™), the leukemia cell lines CCRF-CEM [CCRF CEM] (ATCC® CCL-119™) (CEM) and HL60 (ATCC® CCL-240™), the prostate cancer cell line LNCaP (ATCC® CRL-1740™) and the human embryonic kidney cell line HEK293T (ATCC® CRL-3216™) were obtained from American Type Culture Collection and maintained following the recommendations from ATCC.

### Primary breast cancer cells

Primary breast cancer cells originating from three different patients were obtained from Celther Polska Ltd. Cells were cultured in human EpiCult™-C Human Medium (StemCell technologies) supplemented with 5% fetal bovine serum and 0.48 μg/mL hydrocortisone following the recommendations from Celther. The primary human breast cancer cells CLTH/BC-7 were obtained from a 49 year old human invasive ductual breast carcinoma patient. The primary human breast cancer cells CLTH/BC8 originate from 47 year old individual with invasive ductual breast carcinoma and CLTH/BC9 are taken from a 49 year old patient with invasive breast carcinoma (ER (−); PR (−); HER (+)) All primary cells are positive for MGB-1 which is specific for breast cells, they express an isoform 7 of MUC-1 which is specific for tumor cells.

### cAMP assay

Two days prior determination of agonist-mediated inhibition of forskolin-induced cAMP production, 20.000 c/w BT-474 were seeded in 96w plates in RPMI1640 containing 10% FBS, 4.5 g/l glucose, 2 mM glutamine, 5 mM HEPES and 1 mM sodium pyruvate. One day after seeding, medium was replaced with serum free RPMI1640 with or without 100 ng/μL pertussis toxin and incubated for another 24h, before stimulation with various agonist concentrations (carried out in triplicates) was performed in presence of 10 μM forskolin and 1 mM 3-isobutyl-1-methylxanthine (IBMX). Reactions were stopped by aspiration of media on ice and cells lysis with 20 μl of lysis buffer containing 1 mM IBMX. The cAMP content of cell extracts was determined using the AlphaScreen® cAMP Assay Kit (Perkin Elmer) according to the manufacturers' protocol. From each 96w 5 μl of lysate were transferred to a 384w plate. Acceptor beads (in stimulation buffer without IBMX) and donor beads were added according to the instructions given by the manufacturer.

### ELISA

An indirect cellular ELISA was used to estimate cell surface expression of N-terminal HA-tagged receptor constructs. Briefly, HEK293T cells were seeded into poly-L-lysine coated 48-well plates (8×10^4^ cells per well) and transfected the following day with 0.2 μg DNA, 80 nM siRNA and 0.5 μl Lipofectamine^TM^ 2000 per well according to the manufacturers' protocol. 24h hours after transfection cells were fixed with formaldehyde, without disrupting the cell membrane and incubated in blocking solution (DMEM with 10% FBS) for 1 h at 37°C. Cells were then incubated with anti-HA-peroxidase-labelled high affinity rat monoclonal antibody (3F10, Roche Molecular Biochemicals, Mannheim, Germany). After removal of excess unbound antibody by extensive washing, H_2_O_2_ and ο-phenylenediamine (2.5 mM each in 0.1 M phosphate-citrate buffer, pH 5.0) were added to serve as substrate and chromogen, respectively. After 15 min the enzyme reaction was stopped by adding 1 M H_2_SO_4_ containing 0.05 M Na2SO4, and colour development was measured bichromatically at 492 and 620 nm using an ELISA reader (TECAN Sunrise plate reader).

### siRNA and plasmid transfection

HCAR1 (ID 27198), HCAR2 (ID 338442), HCAR3 (ID 8843) and Universal scrambled negative control trilencer-27 human siRNA were purchased from OriGene (Table S2). One day prior transfection, 15.000 c/w BT-474, 7.500 c/w HCC1954 and 15.000 c/w HCC38 were seeded in 96w plates in RPMI1640 (HyClone) containing 10% FBS (Sigma Aldrich), 4.5 g/l glucose, 2 mM glutamine, 5 mM HEPES, 1 mM sodium pyruvate (Life Technologies). Just before transfection medium was changed to RPMI1640 containing 2.5% FBS, 2 g/l glucose and 2 mM glutamine. 24h before transfection 15.000 c/w MCF12A were seeded in 96w plate in a 1:1 mixture of Dulbecco's modified Eagle's medium (DMEM) and Ham's F12 medium (Life Technologies) supplemented with 5% horse serum (Sigma Aldrich), 20 ng/ml human epidermal growth factor (Sigma Aldrich), 100 ng/ml cholera toxin (Sigma Aldrich), 0.01 mg/ml bovine insulin (Sigma Aldrich) and 500 ng/ml hydrocortisone (Sigma Aldrich). Prior transfection medium was replaced with fresh medium. 5.000 c/w HEK293T cells were seeded in a 96w plate in DMEM (Sigma Aldrich) supplemented with 10% FBS and 24h later, before siRNA transfection medium was changed to DMEM supplemented with 2.5% FBS.

Subsequently, cells were transfected using SAINT-RED (Synvolux Therapeutics) or SAINT-MIX (Synvolux Therapeutics) for siRNA or plasmid transfection following manufacturer's instructions, respectively. BT-474, MCF12A and HEK293T cells were transfected with a final concentration of 60 nM or 80 ng plasmid per well, using 1.2 μl SAINT-RED or SAINT-MIX, respectively. HCC1954 cells were transfected with a final concentration of 40 nM siRNA or 40 ng plasmid per well, using 0.8 μl SAINT-RED or SAINT-MIX, respectively.

### Generation of human HCA_1_, HCA_2_ and HCA_3_ constructs

Primer pairs (Table S4) were used to amplify human HCAR1 (NM_032554.3), human HCAR2 (NM_177551.3) and human HCAR3 (NM_006018.2) from genomic DNA. Genomic DNA samples were prepared from HCC1954 cells using DNeasy®Blood&Tissue Kit (Qiagen). The PCR reaction (50 μl) contained genomic DNA (100 ng) with primers (400 nM each), ThermoPol reaction buffer (1x), dNTP (125 μM, each) and Taq- and Pfu-polymerase (0.5 U each, NEB). The reactions were initiated with a denaturation at 95°C for 2 min, followed by 30 cycles of denaturation at 95°C for 30 s, annealing at 58°C for 30 s and elongation at 72°C for 1 min. A final extension step was performed at 72°C for 10 min. Specific PCR products were directly sub-cloned into the pCR2.1-TOPO vector (Invitrogen) for sequencing. Sequencing reactions were performed with a dye-terminator cycle sequencing kit and applied on an ABI 3700 automated sequencer (Applied Biosystems).

The full length human HCAR1, human HCAR2 and human HCAR3 with an N-terminal hemagglutinin (HA) epitope (YPYDVPDYA) and a C-terminal FLAG-tag (DYKDDDDK) were inserted into the mammalian expression vector pcDNA3.1(−) (Invitrogen).

### Cell viability, apoptosis

ATPlite™ (PerkinElmer) was used to determine cell viability according to manufacturers' instructions. 30 μMetomoxir or 2.5 μM perhexiline were added 6h after transfection and viability was measured 24h (BT-474) or 48h (HCC1954) after transfection (Figure 6B, 6C). Crystal violet staining was used to observe cell viability with microscopy (Figure 4A). 48h after siRNA transfection medium was completely removed from all wells and cells were incubated 10 min with a 1% crystal violet solution in 3.6% paraformaldehyde, 1% MeOH and 20% EtOH. Subsequently, cells were washed three times with water and microscoped. Caspase-Glo® 3/7 Assay (Promega) was used to measure apoptosis. Z-VAD-FMK (Promega) was added to a final concentration of 30 μM 30 min before siRNA transfection and apoptosis was measured 24h (BT-474, HCC38) or 48h (HCC1954) after transfection.

### RNA preparation, reverse transcription and quantitative real-time PCR

Here, we provide a detailed protocol description according to the MIQE guidelines [32] regarding each experimental step as well as choice of reference genes, data analyses and primers used.

To determine basal expression of HCA_1_, HCA_2_ and HCA_3_, BT-474 (500.000 c), HCC1954 (150.000 c), HCC38 (500.000 c), MCF12A (500.000 c), A549 (500.000 c), DMS53 (1.000.000 c), CEM (1.000.000 c) HL60 (1.000.000 c), LNCaP (1.000.000 c) and primary breast cancer (2.000.000 c) cells were seeded in a 6w plate and cultured for 48h in their respective medium (as recommended from ATCC) before harvested for RNA preparation. To determine siRNA knock-down efficiency BT-474 and HCC1954 cells were siRNA transfected in 96w plates as stated before in RPMI1640 containing 2.5% FBS, 2 g/l glucose and 2 mM glutamine (corresponding qPCR data in Figure S4) or RPMI1640 containing 10% FBS, 4.5 g/l glucose, 2 mM glutamine, 5 mM HEPES and 1 mM sodium pyruvate with medium change before and 24h after transfection (corresponding qPCR data in Figure S5E-F), and cells of four wells were harvested 48h after transfection. Total RNA isolation was performed using RNeasy® Mini Kit (Qiagen) following manufacturers' instructions. RNA concentration was determined with a NanoDrop® ND-1000 in TE (pH 8), OD A260/A280 ratios were 1.9-2.0. Prior reverse transcription, 1 μg RNA (determination of basal HCA expression in cell lines and primary breast cancer cells) or 100-300 ng (determination of knock-down efficiency) were treated with 1 μl DnaseI (NEB) in a total reaction volume of 10 μl for 10 min at 37°C. Reaction was stopped by addition of 1 μl 50 mM EDTA and heat inactivation for 10 min at 75°C. Directly thereafter, RNA was reverse transcribed using iScript™ cDNA Synthesis Kit (Bio-Rad) following the manufacturer's instructions. TissueScan^TM^ Cancer Survey cDNA qPCR Array – I (CSRT501) from OriGene Technologies is a panel of normalized cDNA from multiple cancer tissues. A report, including information about disease state for each sample as well as an electropherogramm indicating RNA quality and bioanalyzer ratio is provided (CSRT101 - http://www.origene.com/qPCR/Tissue-qPCR-Arrays.aspx). OriGene received high quality total RNA from Cytomyx and generated first-strand cDNA using an oligo-dT primer. The average amount of cDNA is 2-3 ng per well but can vary due to normalization based on β-actin.

The qPCR setup was as follows: each reaction (20 μl) contained cDNA (10-20 ng for determination of basal expression levels of HCA in cell lines and primary breast cancer cells; 2-5 ng for determination of knock-down efficiency after siRNA transfection), primers (500 nM each in final reaction) and 10 μl iTaq™ Universal SYBR® Green Supermix (Bio-Rad). The following thermal cycling protocol was used: polymerase activation at 95°C for 30 s, followed by 40 cycles of 15 s of denaturation at 95°C and 30 s of monitored annealing/extension at 60°C. Subsequently, melt curves were recorded (55°C - 94.5°C, 0.5 °C increment, 10 s/step). Real-time PCR and data collection (analysis mode: PCR baseline subtracted, baseline was manually set to 500 for cancer panel data and 300 for all other data) were performed on Bio-Rad®iQ™5 Real-Time PCR Detection System.

If not specified differently, primers (stated in Table S5) were designed using Primer-Blast (http://www.ncbi.nlm.nih.gov/tools/primer-blast/) [33], product size: 80-200 bp, Tm 59°C - 61°C, max T_m_ difference 1°C and high primer specificity stringency settings. Primers with highest specificity and lowest values for 3′- and self-complementarity were chosen and ordered from Life Technologies. With each primer pair specified in Table S5 and Table S6, melt curves after the qPCR run showed as one single sharp peak. Agarose gel analyses revealed only one band of correct size. Primer efficiency (stated in Table S5) was determined using 1/5 dilutions (4x) of pooled cDNA (∼20 ng). Data was analyzed using linear regression analysis implemented in GraphPad Prism version 6.03 for Windows (GraphPad Software). Raw C_q_ values collected from the thermocycler were imported and analyzed using the Gene Expression Analysis for iCycler iQ® Real-time PCR Detection System version 1.10 (2004) Excel Spreadsheet that was downloaded from http://www.bio-rad.com/ and is derived from algorithms outlined by Vandesompele et al. [34]. Primer efficiency was set 100%, thus using the ΔΔC_q_ method to analyze the data. TissueScan^TM^ Cancer Survey cDNA qPCR Array data was normalized to endogenous control/reference gene β-actin (n = 2) as recommended from OriGene after consultation of product support, using the primers provided (stated in Table S5). qPCR for each HCA was run in duplicate using commercially available primer (SABiosciences, information provided stated in Table S6) for one replicate and self-designed primer pairs for the second replicate (primer No. 1-6 stated in Table S5). Basal expression of HCA_1_, HCA_2_ and HCA_3_ was normalized to reference gene RPS18. In total, 5 reference genes (RPS18, ACTB, GAPDH, RPL13A, RPLO) were tested and Normfinder [35] was used to identify the most stably expressed gene (stability values: RPS18: 0.104, ACTB: 0.375, GAPDH: 0.174, RPL13A: 0.312, RPLO: 0.174). All qPCR data from knock-down experiments was normalized to RPS18 and ACTB. 5 reference genes were tested for knock-down in HCC1954 and again Normfinder [35] was used to determine stability values (RPS18: 0.050, RPL13A: 0.050, ACTB: 0.060, HSC70: 0.377, H2ai: 0.480). For all experiments indicated n reflect biological replicates, that were reverse-transcribed and qPCR was run in duplicates.

### Liquid Chromatography Mass Spectrometry (LC-MS) measurement

One day prior transfection, 15.000 BT-474 cells per well were seeded in 96w plates in RPMI1640 containing 10% FBS, 4.5 g/l glucose, 2 mM glutamine, 5 mM HEPES, 1 mM sodium pyruvate. Medium was changed prior and 24h after transfection and cells were transfected as described above. 48h after transfection the 96w plates were briefly centrifuged and 80 μl medium were directly added to an Eppendorf tube containing 320 μl ice-cold MeOH. Tubes were incubated 20 min at −20°C, 10 min centrifuged (14.000 rpm) at 4°C and supernatant was transferred to LC-MS glass vials, dried down in a speed vacuum concentrator and stored at −20°C until analysis. Samples were dissolved in 20 μL 50:50 MeOH:H_2_O of which 2 μL were injected into the Agilent 1290 LC-system connected to a 6550 Agilent Q-TOF mass spectrometer and an electrospray ionization (ESI) source was used. Data was collected in positive and negative ionization mode. ESI (Agilent Jetstream) settings were as follows; gas temperature 300°C, gas flow 8 l/min, nebulizer pressure 40 psi, sheet gas temperature 350°C, sheet gas flow 11, Vcap 4000, fragmentor 100, Skimmer1 45 and OctapoleRFPeak 750. Medium metabolites were separated using reverse phase chromatography (Kinetex C18, 100 mm * 2.1 mm, 2.6 μM 100 Å, Phenomenex). For reversed phase elution, solvents were prepared as follows (A) H_2_O, 0.1 % formic acid (B) 75:25 acetonitrile: isopropanol, 0.1% formic acid. All solvents were of HPLC grade. Linear gradients were devised as follows for reversed phase separation (0.5 mL/minute) minute 0: 5%B, minute 8: 95%B, minute 10: 95%B, minute 10.2: 5%B, minute 12: 5%B. Data was analyzed using Mass Hunter Qual (Agilent) using the “find by formula” function with a match tolerance for masses of 10 ppm and for retention times of 0.35 min. The list of all compounds analyzed, including sum formulas, monoisotopic masses and normalized fold changes can be found in Table S3. Most of the metabolites were identified using synthetic standards obtained from Sigma Aldrich, Santa Cruz Biotechnology or Cayman Chemical comparing accurate mass, retention time and in some cases MS/MS spectra.

### Statistical analyses and graphs

All statistical analyses and determination of concentration-response curves of agonists were performed and graphs generated using GraphPad Prism version 6.03 for Windows (GraphPad Software). All indicated n reflect the number of biological replicates.

## SUPPLEMENTARY MATERIAL FIGURES AND TABLES


