# Supplementary material for: Hydroxycarboxylic acid receptors are essential for breast cancer cells to control their lipid/fatty acid metabolism
Source: Oncotarget. 2015 Mar 14;6(23):19706–20. doi: 10.18632/oncotarget.3565 (PMC4637315; doi:10.18632/oncotarget.3565)
Supplement: Supplementary file 1 [file oncotarget-06-19706-s001.pdf]

Hydroxycarboxylic acid receptors are essential for breast cancer cells to control their lipid/fatty acid metabolism

Supplementary Material

Supplemental Tables

Table S1: *Origin and pathology verification of normal tissue samples that are part of the Cancer and Normal TissueScan™ Cancer Survey cDNA qPCR Array – I (CSRT501) as given in the datasheet provided by Origene*

Tissue of (Origin/Finding)	Appearance	Pathology verification notes from H&E review
Breast / Breast	Normal	10% Glandular epithelium, 25% Stroma, 65% Adipose tissue
Breast / Breast	Normal	35% glandular epithelium, 65% stroma
Breast / Breast	Normal	Non Tumor Structures: 20% Glandular epithelium, 20% Stroma, 60% Adipose tissue
Colon: right / Colon: right	Normal	Non Tumor Structures: 5% Mucosa, 50% Submucosa, 45% Muscle
Colon: sigmoid / Colon: sigmoid	Normal	20% Mucosa, 80% Submucosa and Muscle
Colon: transverse / Colon: transverse	Normal	Non Tumor Structures: 20% Mucosa, 20% Submucosa, 60% Muscle
Kidney / Kidney	Normal	100% cortex
Kidney / Kidney	Normal	90% cortex, 10% medulla, 0% pelvis
Kidney / Kidney	Normal	100% cortex
Liver / Liver	Normal	98% lobules, 2% bile ducts
Liver / Liver	Normal	98% Hepatocytes, 2% Portal triads
Liver / Liver	Normal	97% lobules, 3% triads
Lung / Lung	Normal	90% alveoli, 5% bronchioles, 5% fibrovascular septa
Lung / Lung	Normal	80% alveoli, 5% bronchioles, 15% fibrovascular septae; prominent lymphoid aggregates present in fibrous septae
Lung / Lung	Normal	90% alveoli, 10% fibrovascular septae and pleura
Ovary / Ovary	Normal	75% ovarian stroma, 25% hilar vessels
Ovary: right / Ovary: right	Normal	65% ovarian stroma, 5% hilar vessels, 30% corpus luteum

Ovary / Ovary	Normal	95% ovarian stroma, 5% hilar vessels; contains large corpus lutea
Prostate / Prostate	Normal	30% glandular epithelium, 70% fibromuscular stroma
Prostate / Prostate	Normal	20% epithelium, 80% fibromuscular stroma
Prostate / Prostate	Normal	25% epithelium, 70% fibromuscular stroma
Thyroid gland / Thyroid gland	Normal	80% thyroid follicles, 20% fibrovascular stroma/septae
Thyroid gland / Thyroid gland	Normal	Non Tumor Structures: 80% Thyroid follicles, 20% Fibrous stroma
Thyroid gland / Thyroid gland	Normal	85% thyroid follicles, 15% fibrous stroma

Table S2: *siRNA sequences as stated by OriGene*

OriGene Catalog No	gene	sequence
SR309029A	human HCA_1	rGrGrArCrUrCrUrArCrGrArArArUrGrArCrArArGrCrUrGCC
SR309029B	human HCA_1	rGrCrArUrCrArGrUrGrUrGrGrCrArArArUrArGrUrUrUrCCA
SR309029C	human HCA_1	rArGrUrGrGrUrGrArCrUrUrArGrArArUrUrArArCrrUrCrGTG
SR317390A	human HCA_2	rArCrArArCrUrArUrGrUrGrArGrGrCrGrUrUrGrGrGrArCTG
SR317390B	human HCA_2	rGrCrGrArUrUrUrArGrGrGrArArArCrGrGrUrGrGrCrArGAT
SR317390C	human HCA_2	rGrGrCrCrCrArArCrCrUrCrUrCrCrUrUrArArArUrArArCCA
SR305839A	human HCA_3	rGrGrCrArGrGrCrArCrGrGrArArCrCrArCrArCrGrUrUrCAC
SR305839B	human HCA_3	rCrArArCrCrUrCrArArArUrArArCrCrArUrUrCrCrArArGAA
SR305839C	human HCA_3	rGrCrCrGrUrUrCrGrUrGrArUrGrGrArCrUrArCrUrArUrGTG

Table S3: *Targeted list of compounds that were detected in medium of siRNA transfected BT-474 using LC-MS.*

**positive mode**			**metabolite concentration in medium as % of siNC transfected BT-474**					
monoisotopic mass	sum formula	name	$siHCA_3$ (15)	$siHCA_3$ (30)	$siHCA_3$ (60)	siNC	medium	p-value $siHCA_3$ (15) vs siNC
231.1471	C11H21NO4	Isobutyryl-L-carnitine	97.7±1.7	97.7±1.6	96.0±2.0	100	93.6±0.4	ns
231.1471	C11H21NO4	Butyrylcarnitine	100.7±1.8	96.2±1.4	77.3±6.7	100	36.1±0.7	ns
243.1471	C12H21NO4	Tiglylcarnitine	89.7±4.0	82.6±0.1	78.3±0.6	100	57.7±1.5	0.0178
259.1784	C13H25NO4	Hexanoylcarnitine	103.7±3.3	106.3±4.0	98.6±10.5	100	55.5±3.2	ns
273.1940	C14H27NO4	Heptanoylcarnitine	95.3±4.7	98.0±5.1	100.0±5.5	100	107.4±4.7	ns
287.2097	C15H29NO4	Octanoylcarnitine	83.3±18.0	81.3±13.8	64.9±17.6	100	39.2±17.1	ns
315.2410	C17H33NO4	Decanoylcarnitine	122.9±1.6	126.2±8.4	98.2±18.8	100	17.1±1.1	0.0046
343.2723	C19H37NO4	Dodecanoylcarnitine	120.7±2.5	135.2±3.1	127.3±7.8	100	55.1±11.3	0.0143
371.3036	C21H41NO4	Tetradecanoylcarnitine	112.9±2.9	125.5±6.1	122.0±8.9	100	60.7±2.9	0.0470
399.3349	C23H45NO4	Hexadecanoylcarnitine	109.5±5.2	133.1±2.8	116.5±3.4	100	56.6±9.9	ns
425.3505	C25H47NO4	Oleoylcarnitine	124.2±7.0	155.8±1.7	154.1±0.4	100	101.6±10.5	0.0738
427.3662	C25H49NO4	Octadecanoylcarnitine	94.6±3.1	108.9±5.5	103.5±7.4	100	93.4±0.2	ns
**negative mode**								
74.0004	C2H2O3	Glyoxylic acid	118.5±11.3	127.8±3.1	142.6±18.1	100	106.2±14.6	ns
116.0837	C6H12O2	Caproic acid	94.7±0.6	97.6±2.2	106.6±4.3	100	101.8±0.1	0.0116
118.0266	C4H6O4	Succinic acid	100.9±1.9	97.4±0.3	90.0±2.8	100	99.5±10.5	ns
118.0630	C5H10O3	3-Hydroxyvaleric acid	98.1±3.0	98.2±3.4	90.3±0.5	100	88.3±0.9	ns
132.0786	C6H12O3	3-Hydroxyhexanoic acid	92.7±1.9	88.2±1.9	75.6±1.1	100	43.5±0.1	0.0625
134.0216	C4H6O5	Malic acid	116.4±12.4	126.3±4.1	139.8±19.2	100	117.5±4.6	ns
146.0215	C5H6O5	α-Ketoglutaric acid	105.2±5.7	109.3±5.0	103.6±7.8	100	101.4±1.9	ns
160.0735	C7H12O4	Pimelic acid	98.2±1.8	98.4±2.6	105.7±1.6	100	98.6±0.1	ns
160.1099	C8H16O3	3-Hydroxyoctanoic acid	110.4±8.3	104.5±0.8	84.5±3.5	100	NA	ns
165.0790	C9H11NO2	DL-phenylalanine	98.8±0.5	101.4±1.4	104.8±1.2	100	107.3±10.1	ns
166.0630	C9H10O3	3-(3-Hydroxyphenyl)propanoate	88.7±1.2	83.1±0.6	74.1±2.8	100	49.7±3.9	0.0110

174.1117	C6H14N4O2	Arginine	101.3±2.5	99.4±3.4	100.9±2.4	100	105.8±7.0	ns
179.0582	C9H9NO3	Hippurate	96.4±2.2	98.3±0.8	100.3±1.3	100	97.1±0.4	ns
182.0579	C9H10O4	Hydroxyphenyllactic acid	90.9±1.4	89.9±5.2	75.7±0.4	100	60.8±0.6	0.0217
183.0532	C8H9NO4	Pyridoxate	98.4±0.5	99.4±1.1	97.9±3.6	100	95.3±2.1	0.0677
188.1049	C9H16O4	Azelaic acid	91.8±5.6	83.3±3.5	93.7±6.1	100	77.0±9.7	ns
188.1412	C10H20O3	3-Hydroxydecanoic acid	105.5±11.5	112.5±2.5	96.1±7.3	100	NA	ns
192.0270	C6H8O7	Citric acid	105.0±5.9	109.6±5.5	103.9±7.3	100	102.4±1.3	ns
202.1205	C10H18O4	Sebacic acid	87.0±10.8	96.7±3.5	84.8±8.0	100	77.2±12.3	ns
214.1569	C12H22O3	3-Oxododecanoic acid	107.0±6.2	101.5±3.5	88.7±5.7	100	NA	ns
216.1362	C11H20O4	Undecanedioic acid	70.2±17.3	78.5±12.8	51.3±1.7	100	28.1±12.2	ns
216.1725	C12H24O3	3-Hydroxydodecanoic acid	115.3±2.7	116.4±2.3	119.8±0.1	100	NA	0.0298
216.1725	C12H24O3	12-Hydroxydodecanoic acid	68.2±6.0	67.1±7.6	62.0±1.3	100	NA	0.0334
219.1170	C9H17NO5	D-pantothenate	96.8±2.3	99.4±0.5	99.0±2.4	100	98.7±1.0	ns
230.1518	C12H22O4	Dodecanedioic acid	48.9±7.8	59.2±9. 5	35.0±3.6	100	12.8±6.7	0.0222
242.1882	C14H26O3	3-Oxotetradecanoic acid	122.6±1.4	127.7±4.7	122.4±0.5	100	NA	0.0036
244.1675	C13H24O4	1,11-Undecanedicarboxylic acid	54.3±14.3	67.9±2.9	45.6±11.3	100	12.4±9.2	0.0851
256.2402	C16H32O2	Palmitic acid (16:0)	86.6±5.9	96.7±9.7	108.9±12.2	100	109.7±17.3	ns
258.1831	C14H26O4	Tetradecanedioic acid	70.3±6.6	69.5±5.4	56.8±3.7	100	41.1±2.7	0.0465
272.2351	C16H32O3	3-Hydroxy-hexadecanoic acid	97.9±10.3	127.2±3.2	171.0±18.8	100	263.5±12.6	ns
282.2559	C18H34O2	Oleic acid (18:1(n-9))	103.8±9.0	121.7±12.9	153.0±6.6	100	123.8±3.3	ns
284.2715	C18H36O2	Stearic acid (18:0)	89.9±16.9	97.0±22.7	110.1±21.8	100	116.6±28.6	ns
300.2659	C18H36O3	3-Hydroxy-octadecanoic acid	69.2±4.9	57.4±3.9	38.2±1.1	100	NA	0.0243
304.2402	C20H32O2	Arachidonic acid (20:4(n-6))	118.5±0.6	152.2±21.6	219.7±1.2	100	220.7±21.8	0.0011
328.2402	C22H32O2	Docosahexaenoate 22:6(n-3)	104.1±1.5	119.5±0.9	146.7±7.2	100	121.3±22.1	ns
376.1383	C17H20N4O6	Riboflavin (Vitamin B2)	105.0±0.6	103.5±3.6	103.3±6.4	100	113.2±5.1	0.0121
441.1397	C19H19N7O6	Folic acid	98.1±2.0	99.5±1.3	98.5±1.3	100	97.8±1.0	ns
449.3141	C26H43NO5	Glycochenodeoxycholate	92.1±7.3	94.9±2.9	90.6±8.9	100	91.9±7.0	ns
449.3141	C26H43NO5	Glycodeoxycholate	97.0±3.3	94.4±1.3	90.7±8.5	100	93.7±9.0	ns
465.3090	C26H43NO6	Glycocholic acid	93.7±4.4	94.2±4.5	92.1±10.9	100	87.9±3.2	ns
495.3325	C24H50NO7P	LysoPC(16:0)	84.3±0.6	84.8±0.3	92.6±3.2	100	144.5±15.3	0.0012

499.2968	C26H45NO6S	Taurochenodeoxycholate	97.0±1.5	96.9±0.3	90.5±9.3	100	95.8±8.9	ns
499.2968	C26H45NO6S	Taurodeoxycholate	90.1±4.1	88.8±3.2	90.9±6.9	100	92.6±12.5	ns
515.2917	C26H45NO7S	Taurocholate	100.0±0.6	96.6±2.0	93.3±6.8	100	96.6±8.6	ns
521.3481	C26H52NO7P	LysoPC(18:1)	82.0±0.9	82.4±3.9	92.4±9.1	100	259.2±38.7	0.0025
523.3638	C26H54NO7P	LysoPC(18:0)	76.7±6.7	78.3±3.9	85.9±6.5	100	150.8±12.6	0.0350

NA: not detectable in medium alone; ns: not significant $p \geq 0.1$; p-value determined using two-tailed unpaired t-test

Table S4: HCA$_1$, HCA$_2$ and HCA$_3$ cloning primers

**Name**	**Sequence (5'-3')**
GGTGAGTGCTAACGCTCAGAT	HCAR1-ORF-S
CCGACAGAATGAGAAGGATGC	HCAR1-ORF-AS
CCCCGACTACGCCTACAACGGRTCGTGCTG	HCAR1-S-HA-adaptor
CGTCATCGTCCTTATAGTCGTGCCACTCAACAATGT	HCAR1-AS-FLAG-adaptor
CAACACCCTGACATGACATAAAG	HCAR2/3-ORF-S
TCCCTGAGTCCATTTCTGCTAA	HCAR2/3-ORF-AS
CCCCGACTACGCCAATCGGCACCATCTGCAG	HCAR2/3-S-HA-adaptor
CGTCATCGTCCTTATAGTCAGGAGAGGTTGGGCC	HCAR2-AS-FLAG-adaptor
CGTCATCGTCCTTATAGTCCTCGATGCAACAGCC	HCAR3-AS-FLAG-adaptor
CGCGAATTCCCCACCATGTACCCCTACGACGTCCC	HA-S-universal
CGCCGCACTAGTTCACTTATCGTCATCGTCCTTATAGTC	FLAG-AS-universal

Table S5: qPCR primer sequences

No.	Name	Sequence (5'-3')	primer efficiency mean±SEM n=3	GenBank accession number (NCBI)
1	HCAR1-387-forward	GCCCAGCACTGTTTACCTTTTC	1.03±0.02	NM_032554.3
2	HCAR1-496-reverse	CCCCAAAAGCCCAGTGTCTAC		
3	HCAR2-266-forward	TTTCCTGTTCAACCTGGCAGT	0.99±0.02	NM_177551.3
4	HCAR2-352-reverse	CAGTCCCAACGCCTCACAT		
5	HCAR3-1231-forward	GCCCAACCTCAAATAACCATTCC	0.96±0.01	NM_006018.2
6	HCAR3-1316-reverse	CTCGATGCAACAGCCCAACT		
7	HCAR3-86-forward	TACAGACACACGCCACTTTG	n.d.	NM_006018.2
8	HCAR3-246-reverse	GCAACACCTTGGCAATGAAG		
9	HCAR3-460-forward	TGGTGCTCTTCATGTTTGCC	n.d	NM_006018.2
10	HCAR3-626-reverse	GCCAACAGTGATGCCCCA		
11	RPS18-106-forward	GATGGGCGGCGGAAAATAG	0.96±0.02	NM_022551.2
12	RPS18-192-reverse	GTCTGCTTTCCTCAACACCAC		
13	ACTB-1412-forward	ACAATGTGGCCGAGGACTTT	0.95±0.02	NM_001101.3
14	ACTB-1519-reverse	TGGGGTGGCTTTTAGGATGG		
15	RPL13A-179-forward	GTCGTACGCTGTGAAGGCAT	0.97±0.03	NM_012423.3
16	RPL13A-279-reverse	CGGGAAGGGTTGGTGTTCAT		
17	GAPDH-121-forward	CAGTCAGCCGCATCTTCTTTTG	n.d.	NM_002046.4
18	GAPDH-206-reverse	AATCCGTTGACTCCGACCTTC		
19	RPLO-802-forward	GAAACTCTGCATTCTCGCTTCC	n.d.	NM_001002.3
20	RPLO-926-reverse	GCCAGGACTCGTTTGTACCC		
21	HSC70-166-forward	AAACCGAACCACTCCAAGCT	n.d.	AF352832.1
22	HSC70-265-reverse	AACTGTGTTGGTGGGGTTCA		
24	H2ai-425-forward	GCTGCAAACTAATCCAGGCTT	n.d.	NM_003509.2
25	H2ai-440-reverse	CTTTGGGATTGAGTTGCTGCA		
26	ACTB-OriGene forward	CAGCCATGTACGTTGCTATCCAGG	n.d.	NM_001101
27	ACTB-OriGene reverse	AGGTCCAGACGCAGGATGGCATG		

Table S6: SABiosciences qPCR primers

SABiosciences Catalog No	Gene symbol	RefSeq Accession	Band size	Reference position
330001 PPH01865A	HCAR1	NM_032554.3	122	475
330001 PPH58193B	HCAR2	NM_177551.3	182	395
330001 PPH01346E	HCAR3	NM_006018.2	161	1370

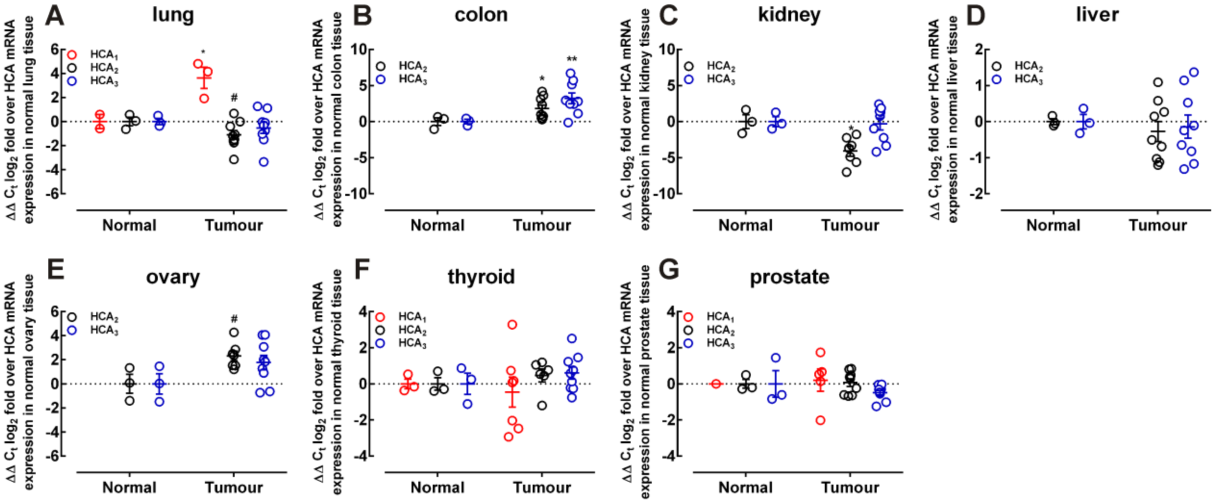

Figure S1: HCA mRNA expression in 7 different cancer tissues versus normal tissues. mRNA expression of HCA_1, HCA_2 and HCA_3 in (A) lung (B) colon, (C) kidney, (D) liver, (E) ovary, (F) thyroid and (G) prostate cancer versus normal patient tissue (two-tailed unpaired t-test, Welch's correction) No HCA_1 mRNA expression was detectable in colon, kidney, liver and ovary normal tissue, but in 3 samples of colon cancer and 4 samples of ovarian cancer patient tissue (data not shown). Significance was assessed using a two-tailed unpaired t-test followed by Welch's correction. # $P \leq 0.1$ * $P \leq 0.05$; ** $P \leq 0.01$; *** $P \leq 0.001$. All data is shown as mean ± SEM. One point reflects one patient tissue sample. Values were determined in technical duplicates.

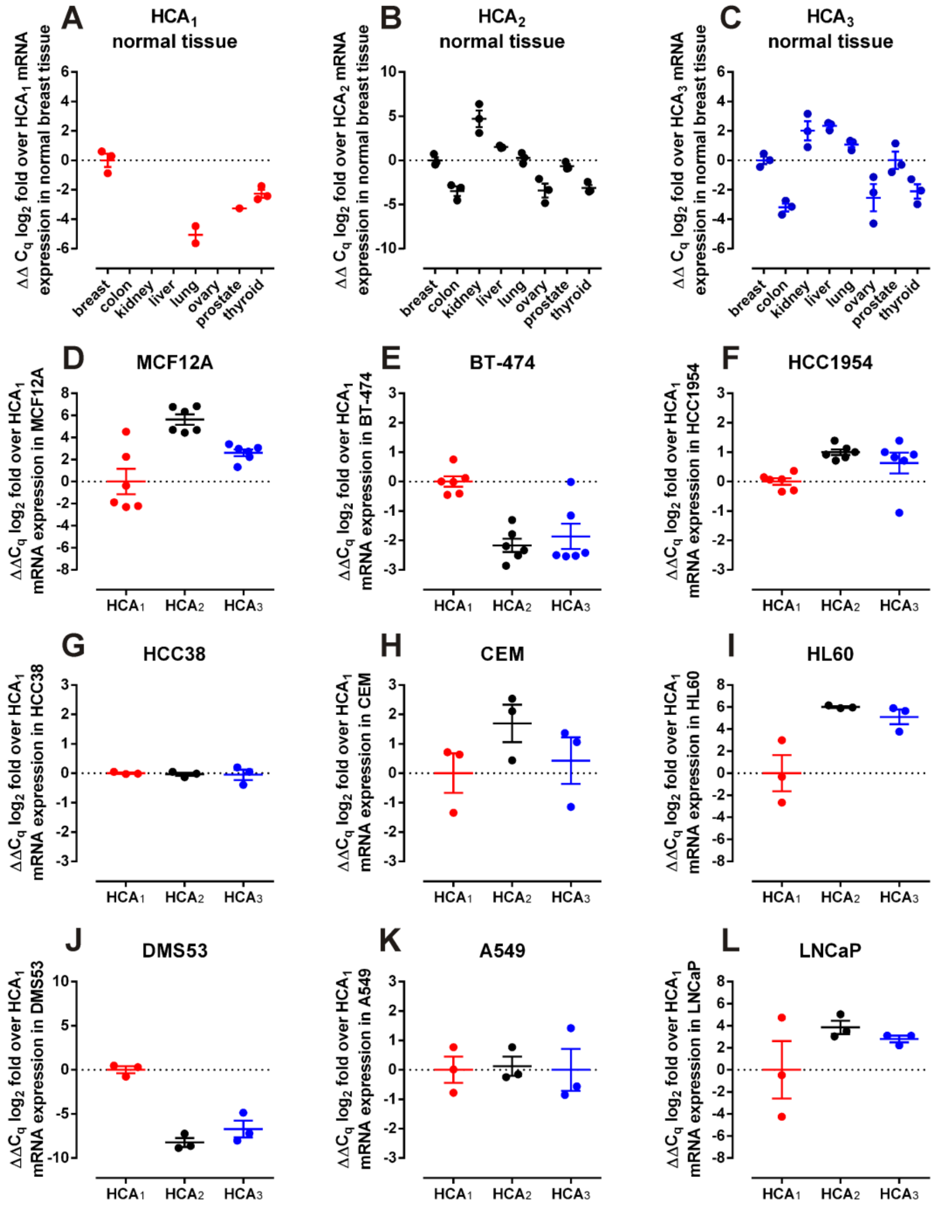

Figure S2: HCA mRNA expression in normal tissue and relative to HCA_1 mRNA expression in 9 cell lines. mRNA expression of (A) HCA_1, (B) HCA_2 and (C) HCA_3in normal patient tissue samples. (A-C) Respective HCA mRNA expression in breast was set 1. (D-L) Presented is HCA_2 and HCA_3 mRNA expression relative to HCA_1 (set 1) in 9 different cell lines. All data is shown as mean ± SEM. Each point reflects a biological replicate.

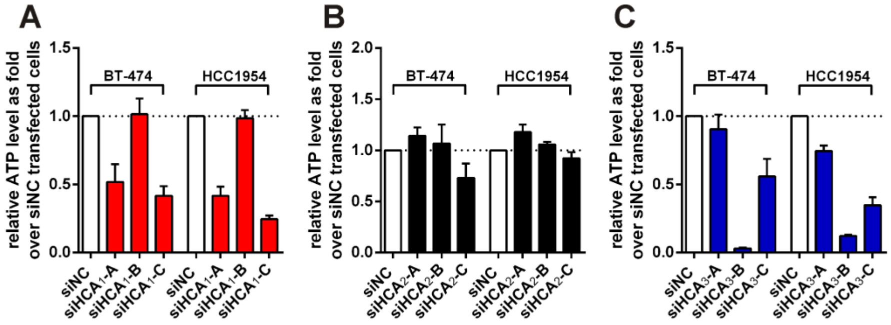

Figure S3: Effect of single siRNAs directed against HCA_1, HCA_2 and HCA_3 on BT-474 and HCC1954 cells. Cell viability of $siHCA_1$-A, $siHCA_1$-B and $siHCA_1$-C (A), $siHCA_2$-A, $siHCA_2$-B and $siHCA_2$-C (B) and $siHCA_3$-A, $siHCA_3$-B and $siHCA_3$-C (C) versus siNC transfected BT-474 and HCC1954 cells was determined 72h after transfection using the ATPlite assay (Perkin Elmer). All data is shown as mean ± SEM of 3 independent experiments carried out in triplicates.

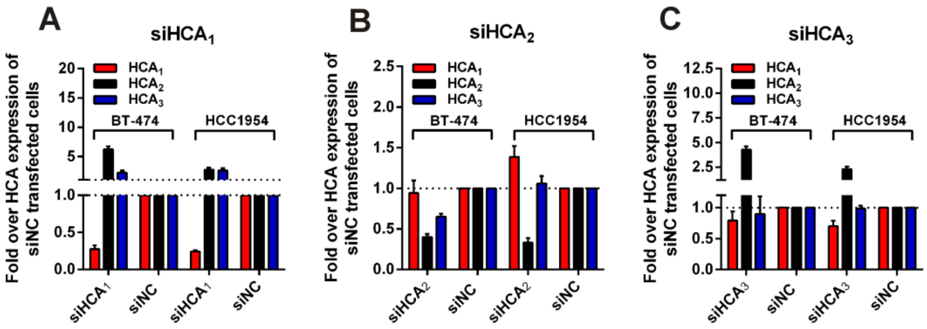

Figure S4: HCA mRNA expression levels in BT-474 and HCC1954 48h after siRNA transfection. (A) 70-80% knock-down of HCA_1 was detectable in both cell lines, whereas HCA_2 and HCA_3 mRNA level was up-regulated. (B) $siHCA_2$ induced 60-70% knock-down of HCA_2. In BT-474 HCA_3 mRNA expression level was about 30% reduced. (C) HCA_3 knock-down was not consistently detectable due to changes in reference genes caused by induced apoptosis. Data (mean ± SEM, n = 6) is shown as fold over expression in siNC-transfected cells.

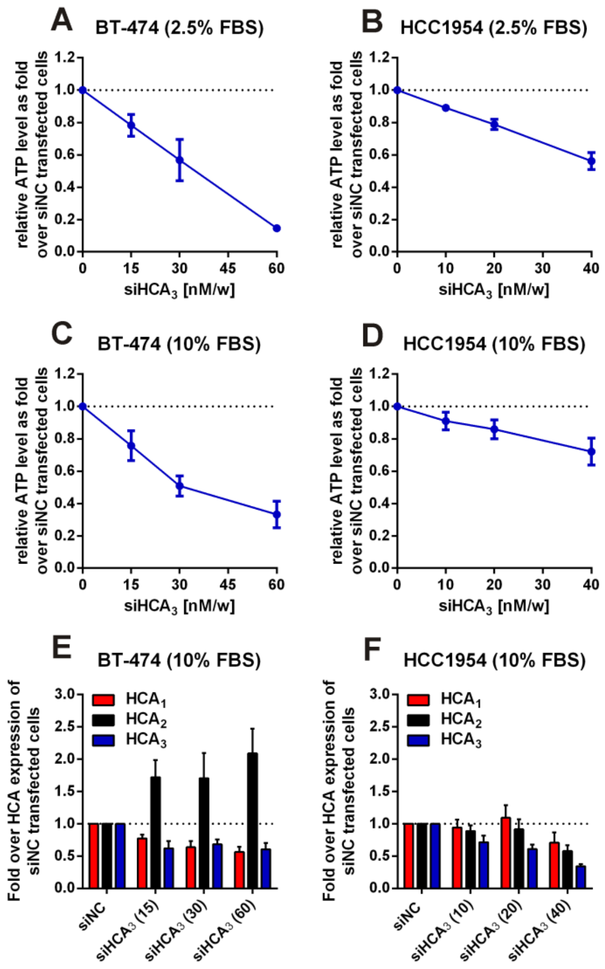

Figure S5: Cell viability and HCA mRNA expression levels in BT-474 and HCC1954 transfected with decreasing concentrations of siHCA$_3$. Cell viability was determined 48h after siRNA transfection carried out in (A, B) RPMI1640 supplemented with 2.5% FBS (n= 2) or (C, D) 10% FBS (n = 3). X-axis shows the amount of HCA3-specific siRNA per well. 50% knock-down of HCA3 was detectable in BT-474 with 60 nM siHCA3 per well (n = 3). (F) In HCC1954 a final concentration of 40 nM siHCA3 per well induced 70-80% knock-down of HCA3 (n = 3). All data is shown as mean ± SEM.

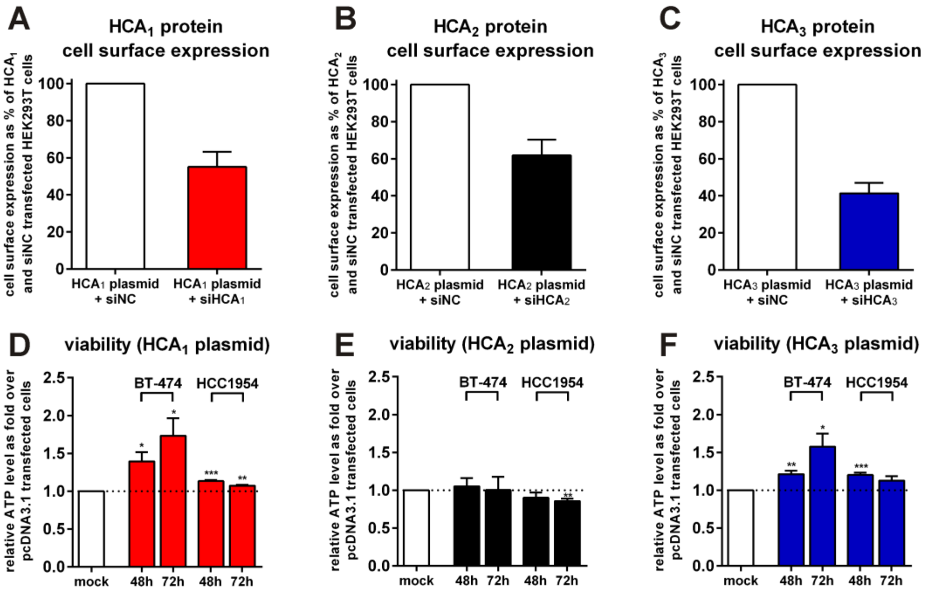

Figure S6: Effect of siRNA-induced knock-down on HCA cell surface expression of transiently with HCA plasmid transfected HEK293T cells and impact of HCA overexpression on BT-474 and HCC1954 viability. Cell surface expression levels of transiently with HA-tagged HCA_1 (A), HCA_2 (B) or HCA_3 (C) in pcDNA3.1 and respective HCA-specific siRNA or siNC co-transfected HEK293T cells were measured by a cell surface ELISA. Specific optical density (OD) readings are given as a percentage of HEK293T cells co-transfected with respective HA-tagged HCA encoding plasmid and siNC. The non-specific OD value (empty vector) was 0.001 ± 0.0003. The OD value of the HA-tagged HCA_1 was 0.010 ± 0.002 (A), of the HA-tagged HCA_2 was 0.431 ± 0.044 (B) and HA-tagged HCA_3 was 0.197 ± 0.034 (C). Data is presented as mean ± SEM of 3 independent experiments carried out in triplicates. Cell viability of transiently with HCA_1 in pcDNA3.1 (D), HCA_2 in pcDNA3.1 (E) or HCA_3 in pcDNA3.1 (F) transfected BT-474 and HCC1954 cells was determined 48h or 72h after transfection using the ATPlite assay (Perkin Elmer). (D-F) Data is shown as mean ± SEM of 4 independent experiments carried out in triplicates. P values were determined using a two-tailed unpaired t-test. * $P \leq 0.05$; ** $P \leq 0.01$; *** $P \leq 0.001$.
